# Diaspora of oral maxillofacial pathology and dermatopathology-A review

**DOI:** 10.6026/973206300223036

**Published:** 2026-05-31

**Authors:** Pazhanivel Kandasamy, Maimoona Abdul Khader, Barun Kumar Sharma, Shanawaz Abdul Kader Hakam, Sruti Murali, Karthik Shunmugavelu

**Affiliations:** 1 Department of Oral Medicine and Radiology, Thai Moogamabigai Dental College and Hospital, Chennai, Tamilnadu, India; 2 Department of Orthodontics and Dentofacial Orthopedics MES Dental College, Perithalmanna, Malappuram, Kerala, India; 3 Department of Oral and Maxillofacial Surgery, Bharati Vidyapeeth (Deemed to be University) Dental College and hospital Navi Mumbai, India; 4 Private practitioner, Safa Al Bawadi Medical Complex, Jeddah, Saudi Arabia; 5 Private Practitioner, independent researcher and oral and maxillofacial pathologist, Vijaya Dental Care and Implant Centre, Chennai, Tamil Nadu, India; 6 Department of Dentistry, PSP Medical College Hospital and Research Institute, Tambaram, Kanchipuram, Tamilnadu 631604, India

**Keywords:** Oral mucosa, oral lesions, mucocutaneous disorders, dermatology, systemic diseases, sentinel lesions

## Abstract

The oral cavity is an important diagnostic window because it frequently reflects dermatological, infectious, autoimmune, genetic and systemic disorders. Oral lesions may precede cutaneous or systemic manifestations in several conditions, including lichen planus, pemphigus vulgaris, viral infections, connective tissue disorders, vasculitic syndromes and immunodeficiency-associated diseases. The clinical appearance of oral lesions is often modified by salivation, mastication, microbial colonization, trauma and the thin nature of non-keratinized mucosa. Therefore, lesions that appear as vesicles or bullae on skin may present intraorally as erosions, ulcers, pseudomembranes, or erythematous areas. Recognition of these oral "sentinel" lesions is clinically important because early diagnosis can improve patient outcomes, guide systemic evaluation and facilitate timely referral. Thus, we review the clinicopathological basis of oral-cutaneous correlation and summarize important infectious, autoimmune, vesiculobullous, vasculitic, genetic, emergency, granulomatous and histiocytic disorders with oral manifestations. Hence, a multidisciplinary approach involving dentists, dermatologists, physicians and pathologists is essential for accurate diagnosis and comprehensive management.

## Background:

The oral cavity is a highly specialized mucosal environment that frequently reflects local, dermatological and systemic disease. Oral mucosal lesions may present as abnormal growths, ulcerations, vesicles, bullae, white or red patches, swelling, pigmentation, or structural changes affecting the tissues of the oral cavity [1]. Because the oral mucosa and skin share embryological and histological similarities, many dermatological disorders also produce characteristic intraoral changes [2]. However, oral findings are often overlooked, particularly when patients initially present to medical or dermatological services with cutaneous complaints. This is clinically significant because oral lesions may represent the earliest visible manifestation of an infection, autoimmune disorder, immunodeficiency state, systemic illness, or potentially malignant condition [3]. The oral cavity differs from skin in several important ways. Continuous salivation, masticatory trauma, variable keratinization, rich vascularity and diverse microbial colonization influence the morphology and progression of lesions. Consequently, diseases that form intact vesicles or bullae on skin may appear as erosions, ulcers, or pseudomembranous areas in the mouth. These alterations can obscure classic dermatological features and complicate diagnosis [4]. Therefore, accurate interpretation requires careful assessment of lesion site, distribution, symmetry, duration, pain, recurrence, surface morphology, systemic symptoms, immune status, medication history and associated cutaneous findings. Oral lesions can act as sentinel findings in many systemic diseases. In conditions such as pemphigus vulgaris, oral lesions may precede cutaneous involvement by weeks or months. In immunocompromised states, recurrent candidiasis, oral hairy leukoplakia, or severe periodontal disease may indicate declining immune function. In children, strawberry tongue, fissured lips and diffuse oral erythema may provide important diagnostic clues in Kawasaki disease. Similarly, recurrent oral aphthae may be one of the earliest and most consistent features of Behçet's disease [5]. Thus, oral examination should be considered a routine component of dermatological and systemic assessment.

## Clinicopathological basis of oral-cutaneous correlation:

The relationship between oral mucosa and skin provides the biological foundation for oral-cutaneous correlation. Both tissues consist of stratified squamous epithelium supported by connective tissue, but site-specific differences in keratinization, moisture, trauma exposure and microbial environment produce important clinical variations [6]. Keratinized oral sites such as the hard palate and gingiva may show plaques, papules, or keratotic changes, whereas non-keratinized mucosa such as the buccal mucosa, labial mucosa, floor of mouth and soft palate are more prone to erosions and ulceration. The same disease process may therefore appear differently depending on the site involved. In vesiculobullous disorders, fragile blisters often rupture rapidly inside the mouth, leaving irregular erosive surfaces. In viral infections, vesicles may be transient and replaced by painful shallow ulcers. In autoimmune and inflammatory diseases, reticular white striae, erythematous patches, desquamative gingivitis, or chronic ulcers may be the dominant findings [7]. Specific clinical clues remain valuable in narrowing the diagnosis. Wickham striae suggest oral lichen planus, Nikolsky positivity supports pemphigus vulgaris or related blistering disorders, shaggy white plaques on the lateral tongue suggest oral hairy leukoplakia and strawberry tongue is a classic sign in Kawasaki disease [8]. Because many mucocutaneous diseases share overlapping clinical patterns, oral lesions should never be interpreted only by surface appearance. Correlation with systemic symptoms, skin findings, histopathology, immunofluorescence, microbiological tests, serology and radiographic findings may be required for definitive diagnosis [9].

## Infectious diseases with oral manifestations:

## Herpes simplex virus infection:

Herpes simplex virus infection is one of the most common viral causes of painful oral ulceration. Primary herpetic gingivostomatitis is usually seen in children and may present with fever, malaise, cervical or submandibular lymphadenopathy, gingival inflammation and multiple painful oral ulcerations. The lesions may involve the labial mucosa, buccal mucosa, tongue, gingiva and palate. Vesicles are often short-lived and rupture quickly to form shallow ulcers covered by a yellowish pseudomembrane [10]. Recurrent infection most commonly appears as herpes labialis near the vermilion border and may be precipitated by stress, sunlight, fever, trauma, or systemic illness. Clinical recognition is important because herpetic lesions may be confused with aphthous ulcers, erythema multiforme, traumatic ulcers, or other acute ulcerative disorders. Distribution, recurrence pattern, prodromal burning or tingling and systemic symptoms help distinguish herpes simplex infection from other causes of oral ulceration [11].

## Varicella-zoster virus infection:

Varicella-zoster virus produces two clinically distinct patterns: primary varicella and herpes zoster. In varicella, oral lesions may occur along with generalized skin eruptions and are commonly seen on the palate. These lesions begin as fragile vesicles and rapidly ulcerate. In herpes zoster, painful unilateral lesions follow the distribution of an affected sensory nerve. When trigeminal branches are involved, oral lesions characteristically remain unilateral and stop at the midline [12]. Mandibular nerve involvement may affect the tongue and floor of mouth, whereas maxillary division involvement may involve the palate, tonsillar region, or uvula. Oral zoster is clinically important because severe, recurrent, or atypical disease may suggest immunosuppression. It may also be followed by post-herpetic neuralgia, which can cause persistent pain and significant morbidity [13].

## Enteroviral infections:

Enteroviral infections also produce characteristic oral lesions, particularly in children. Herpangina, commonly caused by coxsackie viruses, affects the posterior oral cavity, including the soft palate, tonsillar pillars, uvula and pharynx. Small vesicles surrounded by erythematous halos rupture to form painful ulcers. Hand, foot and mouth disease is associated with coxsackievirus and enterovirus strains and produces oral vesicles and ulcers along with lesions on the hands, feet and sometimes buttocks [14]. These infections usually have a short febrile course but should be differentiated from herpes simplex infection, aphthous disease and immune-mediated ulcerative disorders.

## HIV-associated oral lesions:

Oral lesions are highly significant in human immunodeficiency virus infection because they may reflect immune deterioration. Common HIV-associated oral conditions include candidiasis, oral hairy leukoplakia, Kaposi's sarcoma, recurrent herpes infection, aphthous-like ulcers, and necrotizing periodontal disease and salivary gland disorders [15]. In advanced immunosuppression, otherwise benign infections may become extensive, persistent, recurrent, or resistant to routine therapy. Oral hairy leukoplakia is strongly associated with Epstein-Barr virus infection in immunocompromised individuals. It classically appears as white corrugated or shaggy plaques on the lateral borders of the tongue and is usually asymptomatic. Unlike candidiasis, the lesion cannot be wiped off easily [16]. Identification of oral hairy leukoplakia should prompt assessment of immune status when the underlying cause is not already known.

## Oral candidiasis:

Oral candidiasis is one of the most common opportunistic infections of the oral cavity. It may present as pseudomembranous candidiasis, erythematous candidiasis, denture stomatitis, angular cheilitis, median rhomboid glossitis, or chronic multifocal candidiasis [17]. Pseudomembranous candidiasis presents as white creamy plaques that can often be wiped off, leaving an erythematous or bleeding surface. Erythematous candidiasis may cause burning sensation, soreness, altered taste and mucosal redness. Predisposing factors include diabetes mellitus, immunosuppression, xerostomia, corticosteroid therapy, broad-spectrum antibiotics, denture use, poor oral hygiene, nutritional deficiency and systemic illness [18]. Chronic mucocutaneous candidiasis may involve the oral mucosa, skin, nails and endocrine organs, reflecting an underlying immune defect rather than simple local overgrowth. Recurrent or atypical candidiasis should therefore prompt evaluation for local and systemic risk factors [19].

## Leishmaniasis:

Leishmaniasis is a protozoal infection caused by Leishmania species and transmitted by sandflies. Mucocutaneous leishmaniasis may involve the oral and nasal mucosa in addition to skin. Oral lesions may begin as lip swelling, nodules, or chronic ulcers and can progress to destructive lesions involving the palate, nasal cavity and surrounding tissues [20]. Advanced disease may produce severe facial disfigurement. Although uncommon in routine dental practice, leishmaniasis should be considered in patients from endemic areas or those with relevant travel history, especially when oral ulcers are chronic, destructive, or non-healing [21].

## Syphilis:

Syphilis is an important multisystem bacterial infection with several oral manifestations. In secondary syphilis, oral mucous patches, snail-track ulcers and shallow erosive lesions may occur. These lesions can mimic aphthous ulcers, traumatic ulcers, candidiasis, or inflammatory mucosal disease. In tertiary syphilis, gummatous lesions may cause palatal destruction and oronasal communication. Congenital syphilis may produce characteristic dental abnormalities such as Hutchinson incisors and mulberry molars [22]. Because oral syphilis may clinically resemble other ulcerative diseases, serological testing should be considered in unexplained persistent mucous patches or suspicious oral ulcers.

## Leprosy:

Leprosy, caused by Mycobacterium leprae, may involve the oral cavity, particularly in advanced lepromatous disease. Oral lesions may occur on the palate, gingiva, tongue and uvula. Nodules, ulceration, fibrosis and sensory impairment may be seen. Neural involvement increases the risk of trauma and secondary infection [23]. In endemic regions, chronic nodular or ulcerative oral lesions should be interpreted along with cutaneous lesions, peripheral nerve thickening, sensory loss and systemic findings.

## Connective tissue and autoimmune disorders:

## Lupus erythematosus:

Lupus erythematosus may present with oral lesions in both systemic and chronic cutaneous forms. Lesions may involve the palate, buccal mucosa, lips and gingiva. Clinically, they may appear as erythematous plaques, purpuric areas, erosions, or painful ulcers with a red halo and yellowish base [24]. Lip involvement may present as thickening, crusting, or inflammation. Oral lupus lesions may resemble oral lichen planus, particularly when white striae or erosive areas are present. Differentiation from oral lichen planus requires attention to distribution, symmetry, systemic features, photosensitivity, serological findings and histopathology. Lupus-related oral lesions may be less symmetrical and may extend to the vermilion border, whereas classic oral lichen planus often shows bilateral reticular lesions on the buccal mucosa [25].

## Systemic sclerosis:

Systemic sclerosis affects the oral cavity through fibrosis, vascular compromise, xerostomia and skeletal changes.Clinically, patients may show microstomia, reduced mouth opening, taut facial skin, thin lips, xerostomia and difficulty maintaining oral hygiene. Reduced manual dexterity due to skin tightening may further compromise plaque control [26]. Radiographic changes may include widening of the periodontal ligament space along the full root length and mandibular resorption involving the angle, condyle, coronoid process, or ramus [27]. These findings may complicate dental treatment, prosthodontic rehabilitation and periodontal care.

## Sjögren's syndrome:

SjÖgren's syndrome is an autoimmune disorder primarily affecting exocrine glands, leading to salivary and lacrimal hypofunction. Oral manifestations include xerostomia, burning sensation, difficulty swallowing, altered taste, fissured tongue, rampant dental caries, cervical caries, mucosal soreness and increased susceptibility to candidiasis [28]. Salivary gland enlargement may also occur. Because dryness is a central feature of disease burden, dentists play an important role in identifying patients with persistent xerostomia and referring them for systemic evaluation.

## Vesiculobullous disorders:

## Pemphigus vulgaris:

Pemphigus vulgaris is a serious autoimmune blistering disease characterized by loss of epithelial cell adhesion. Oral lesions commonly precede skin involvement and may persist for months before diagnosis. Because intraoral bullae rupture easily, clinicians usually observe painful irregular erosions, ulcers, or raw mucosal areas rather than intact blisters [29]. Common sites include the buccal mucosa, palate, lips, tongue and gingiva. Desquamative gingivitis may also occur. Nikolsky sign may be positive and diagnosis requires histopathological examination and direct immunofluorescence. Early recognition is essential because delayed diagnosis can result in prolonged pain, difficulty eating, secondary infection and systemic complications [30].

## Paraneoplastic pemphigus:

Paraneoplastic pemphigus is a severe autoimmune mucocutaneous disease associated with underlying neoplasms such as lymphoma, leukemia, carcinoma, sarcoma, or thymoma. Oral lesions are often extensive, painful, refractory and clinically severe. They may present as persistent stomatitis, erosions, ulceration and hemorrhagic crusting of the lips [31]. Because oral lesions may precede recognition of the associated malignancy, severe treatment-resistant stomatitis should raise suspicion for paraneoplastic pemphigus, particularly when accompanied by polymorphous skin eruptions or systemic symptoms [32].

## Mucous membrane pemphigoid:

Mucous membrane pemphigoid is a chronic autoimmune subepithelial blistering disorder that primarily affects mucous membranes. Oral involvement may include desquamative gingivitis, erosions, bullae and ulcerations. Unlike pemphigus vulgaris, mucous membrane pemphigoid may heal with scarring, especially in ocular, pharyngeal and genital sites [33]. Accurate diagnosis and long-term monitoring are important because ocular involvement may lead to serious complications, including symblepharon and blindness.

## Epidermolysis bullosa:

Epidermolysis bullosa comprises inherited disorders characterized by epithelial fragility and trauma-induced blistering. Oral manifestations include recurrent blistering, ulceration, scarring, ankyloglossia, microstomia, vestibular obliteration, enamel defects, dental caries and difficulty maintaining oral hygiene [34]. Severe forms may cause nutritional compromise and functional impairment. Dental care must be preventive, atraumatic and multidisciplinary, with emphasis on minimizing tissue trauma and maintaining oral function [35].

## Oral lichen planus:

Oral lichen planus is a chronic immune-mediated mucocutaneous disorder that may occur with or without skin lesions. Oral lesions may be reticular, papular, plaque-like, atrophic, erosive, bullous, or desquamative. The reticular form is the most characteristic and presents as bilateral lace-like white lines called Wickham striae, commonly involving the buccal mucosa [36]. Erosive and atrophic forms may cause pain, burning sensation, ulceration and difficulty eating. Oral lichen planus can resemble lupus erythematosus, lichenoid drug reactions, contact reactions, graft-versus-host disease and oral potentially malignant disorders. Therefore, persistent erosive, ulcerative, or plaque-like lesions require clinical monitoring and biopsy when indicated [37].

## Contact and hypersensitivity-related oral lesions:

Contact stomatitis and contact cheilitis may occur due to hypersensitivity reactions to foods, flavoring agents, dental materials, mouthwashes, topical medications, cosmetics, or lip products. Clinical features range from erythema and edema to keratotic white plaques, shaggy mucosal changes, erosions, or burning sensation [38]. Lesions may involve the lips, buccal mucosa, tongue margins, or gingiva depending on the exposure. Diagnosis is often difficult because contact reactions can mimic candidiasis, frictional keratosis, lichenoid reactions, or inflammatory mucosal disorders. A detailed history of dental materials, restorations, prostheses, oral hygiene products, cosmetics and topical medications is essential. Improvement after withdrawal of the suspected allergen supports the diagnosis [39].

## Vasculitic and multisystem syndromes:

## Behçet's disease:

BehÇet's disease is a chronic multisystem vasculitic disorder characterized by recurrent oral aphthous ulcers, genital ulcers, ocular inflammation, skin lesions and pathergy. Oral ulcers are among the most common and earliest manifestations. They may be minor, major, or herpetiform and are often painful and recurrent [40]. Because recurrent oral ulceration is common in the general population, suspicion for Behçet's disease increases when ulcers are severe, frequent, scarring, or associated with genital ulceration, ocular symptoms, skin lesions, arthritis, vascular events, or neurological involvement [41].

## Kawasaki disease:

Kawasaki disease is an acute vasculitis of childhood with important mucocutaneous findings. Oral features include diffuse erythema of the oral mucosa, fissured lips, cracked and bleeding lips and strawberry tongue [42]. These findings occur along with persistent fever, bilateral conjunctival injection, cervical lymphadenopathy, extremity changes and polymorphous rash. Early recognition is essential because untreated Kawasaki disease may lead to coronary artery aneurysms and long-term cardiac complications [43].

## Granulomatosis with polyangiitis:

Granulomatosis with polyangiitis, previously called Wegener's granulomatosis, is a necrotizing granulomatous vasculitis affecting the upper respiratory tract, lower respiratory tract, kidneys, skin and oral cavity. Oral lesions may include ulcers, petechiae, mucosal erosions and the classic appearance of strawberry gingivitis [44]. Strawberry gingivitis may precede systemic diagnosis and should prompt urgent evaluation when associated with nasal symptoms, respiratory complaints, renal involvement, constitutional symptoms, or purpuric skin lesions [45].

## Genetic and developmental disorders:

## Tuberous sclerosis complex:

Tuberous sclerosis complex is a genetic multisystem disorder associated with cutaneous angiofibromas, hypopigmented macules, periungual fibromas, seizures, neurodevelopmental abnormalities, renal lesions and other systemic features. Oral manifestations include gingival fibromas, fibromatous growths on the palate and enamel pits [46]. Although these oral findings are often benign, they may contribute to syndromic recognition, especially when combined with characteristic cutaneous or neurological features [47].

## Ectodermal dysplasia:

Ectodermal dysplasias are congenital disorders affecting structures derived from ectoderm, including teeth, hair, nails, skin and sweat glands. Oral findings include hypodontia, anodontia, delayed tooth eruption, peg-shaped teeth, conical teeth, reduced alveolar ridge development and impaired occlusion [48]. These abnormalities affect mastication, speech, facial growth, esthetics and psychosocial well-being. Long-term dental management often requires preventive care, prosthodontic rehabilitation, orthodontic planning and growth-related modifications [49].

## Severe mucocutaneous reactions and emergency conditions:

## Erythema multiforme, stevens-johnson syndrome and toxic epidermal necrolysis:

Erythema multiforme, Stevens-Johnson syndrome and toxic epidermal necrolysis represent clinically important mucocutaneous reaction patterns, often triggered by infection or drugs. Oral involvement may be severe and may include hemorrhagic crusting of lips, widespread erosions, painful ulceration, pseudomembranes and extensive epithelial sloughing [50]. Differentiation among these conditions is essential because prognosis, systemic involvement and management differ significantly. Severe oral ulceration associated with skin detachment, fever, malaise, ocular involvement, genital lesions, or systemic toxicity should be treated as a medical emergency. A careful drug history, recent infection history, extent of mucosal involvement and percentage of skin detachment help guide diagnosis and management [51].

## Angioedema:

Angioedema is a potentially life-threatening condition characterized by rapid swelling of deeper tissues. Oral and oropharyngeal involvement may affect the lips, tongue, floor of mouth, soft palate, uvula and airway. Causes include allergic reactions, medications such as angiotensin-converting enzyme inhibitors, hereditary C1-inhibitor deficiency and idiopathic mechanisms [52]. Unlike superficial urticaria, angioedema may progress rapidly and compromise the airway. Therefore, sudden swelling of the tongue, floor of mouth, or oropharynx requires urgent airway assessment before routine dental or oral evaluation.

## Granulomatous and histiocytic disorders:

## Sarcoidosis:

Sarcoidosis is a multisystem granulomatous disease of unknown etiology that most commonly affects the lungs but may also involve the oral cavity and salivary glands. Oral lesions may present as nodules, swelling, ulcers, gingival enlargement, buccal mucosal lesions, tongue lesions, or parotid enlargement [53]. Because oral sarcoidosis is uncommon and clinically nonspecific, biopsy is usually required. Demonstration of non-caseating granulomatous inflammation and exclusion of infections such as tuberculosis and fungal disease are important before attributing oral lesions to sarcoidosis.

## Langerhans cell histiocytosis:

Langerhans cell histiocytosis may involve bone, skin, lymph nodes and oral tissues. Oral manifestations include gingival enlargement, painful ulcers, premature tooth mobility, periodontal destruction and alveolar bone loss. Radiographically, the teeth may appear to be floating because of severe alveolar bone destruction. These findings may mimic aggressive periodontitis, but suspicion should increase when the destruction is disproportionate to local plaque deposits, occurs in young patients, or is associated with skin lesions, bone pain, or systemic symptoms. Early diagnosis is important because oral lesions may be part of multisystem disease.

## Diagnostic approach and clinical implications:

A systematic approach is essential when evaluating oral mucosal lesions. The clinician should assess onset, duration, recurrence, number of lesions, site, symmetry, pain, color, surface texture, ulcer depth, induration, bleeding, relation to trauma, prosthesis use, drug history, immune status, systemic symptoms and associated skin findings. Lesions may be broadly categorized as ulcerative, vesiculobullous, white, red, pigmented, swelling-type, granulomatous, or mixed-pattern lesions. Persistent ulcers, indurated lesions, unexplained white or red patches, recurrent multifocal erosions, destructive lesions, lesions associated with systemic symptoms and lesions occurring in immunocompromised patients require prompt investigation. Biopsy, cytology, direct immunofluorescence, microbiological tests, serology, imaging and specialist referral may be needed depending on the suspected diagnosis. The clinical importance of oral lesions lies in their ability to reveal hidden systemic disease. A dermatologist may focus on skin findings while missing early oral clues and a dentist may treat oral symptoms locally without recognizing systemic significance. This disciplinary separation may delay diagnosis of serious conditions such as pemphigus vulgaris, lupus erythematosus, Behçet's disease, HIV-associated disease, granulomatosis with polyangiitis, sarcoidosis, or potentially malignant oral disorders. A coordinated approach involving dentists, dermatologists, physicians, pediatricians, rheumatologists, infectious disease specialists and pathologists is therefore essential.

## Conclusion:

The oral cavity is an important diagnostic window for dermatological and systemic diseases. Infectious disorders, autoimmune connective tissue diseases, vesiculobullous disorders, vasculitic syndromes, genetic conditions, severe mucocutaneous reactions, granulomatous diseases and histiocytic disorders may all produce oral manifestations. In several conditions, oral lesions may precede skin or systemic findings and may therefore serve as sentinel signs. Careful oral examination, clinicopathological correlation, timely biopsy and multidisciplinary referral are essential for accurate diagnosis and effective management. Thus, recognition of oral manifestations should be considered an integral part of both dental and medical practice.
